# Is It Time to Move Beyond TIR to TITR? Real-World Data from Over 20,000 Users of Continuous Glucose Monitoring in Patients with Type 1 and Type 2 Diabetes

**DOI:** 10.1089/dia.2023.0565

**Published:** 2024-02-01

**Authors:** Timothy C. Dunn, Ramzi A. Ajjan, Richard M. Bergenstal, Yongjin Xu

**Affiliations:** ^1^ Clinical Affairs, Abbott Diabetes Care, Alameda, California, USA.; ^2^ The LIGHT Laboratories, Leeds Institute of Cardiovascular and Metabolic Medicine, University of Leeds, Leeds, United Kingdom.; ^3^ International Diabetes Center, HealthPartners Institute, Minneapolis, Minnesota, USA.

**Keywords:** Time in range, Time in tight range, Continuous glucose monitoring

## Abstract

The growing use of continuous glucose monitoring (CGM) has been supported by expert consensus and clinical guidelines on glycemic management in diabetes with time in range (TIR 70–180 mg/dL) representing a key CGM-derived glucose metric. Time in tight range (TITR) has also been proposed for clinical use, spanning largely normal glucose levels of 70–140 mg/dL. However, keeping such narrow glucose ranges can be challenging, and understanding the factors modulating TITR can help achieve these tight glycemic targets. Our real-life study aimed to evaluate the relationship between average glucose (AG) and TIR/TITR in a large cohort (*n* = 22,006) of CGM users, divided into four groups: self-identified as having type 1 diabetes (T1D) treated with insulin using multiple daily injections (MDI) or pumps; type 2 diabetes (T2D) on MDI or insulin pumps; T2D on basal insulin only; and T2D not on insulin treatment. The T2D groups, regardless of treatment type, displayed the highest TIR and TITR values, associated with lowest glycemic variability measured as glucose coefficient of variation (CV; 23–30%). The T1D group showed the lowest TIR and TITR, associated with the highest CVs (36–38%). Overall, higher CV was associated with lower TIR and TITR for AG values below 180 and 140 mg/dL, respectively, with the reverse holding true for AG values above these thresholds. The discordance between AG and TIR/TITR was less pronounced in T2D compared with T1D, attributed to lower CV in the former group. It was also observed that TITR has advantages over TIR for assessing glycemia status and progress toward more stringent A1C, particularly when approaching normal glucose levels. The data detail how CV affects the AG relationship with TIR/TITR, which has implications for CGM interpretation. In many instances TITR, rather than TIR, may be preferable to employ once AG falls below 140 mg/dL and near-normal glucose levels are required clinically.

## Introduction

Measurement of blood glucose and glycated hemoglobin (A1C) are clinically used for the diagnosis of diabetes^1^ and to guide management decisions.^2^ More recently, interstitial glucose measurement by continuous glucose monitoring (CGM) has been adopted to guide therapy changes in diabetes, employing a number of glucose metrics, including time in range (TIR), time below range (TBR), and time above range (TAR). In recent studies, TIR has been associated with diabetes complications and outcomes.^3,4^ The association was found to be complementary or independent of A1C,^5^ further emphasizing the value of TIR.

International expert consensus has defined glycemic metrics derived from CGM, including TIR (70–180 mg/dL), TAR (>180 mg/dL), and TBR (<70 mg/dL).^6^ However, since a recent study^7^ utilizing current CGM technology, with a median of 96% time in tight range (TITR) 70–140 mg/dL, a tighter range of 70–140 mg/dL could be measured when achieving near-normal glucose levels is the goal. TITR was identified and recommended recently as an important additional CGM-derived glucose metric covering glucose ranges of 70–140 mg/dL^8,9^ and which can be considered as an alternative to TIR according to study and participant management goals.

Only recently has the relationship between TIR and TITR begun to be carefully analyzed,^10,11^ but there are still limited reports of large data sets from real-life settings, and particularly lacking are data in noninsulin-treated type 2 diabetes (T2D). Therefore, this study aimed to evaluate real-life glucose metrics—with a particular focus on TIR 70–180 mg/dL (TIR) and TITR 70–140 mg/dL (TITR)—in a large cross-section of CGM users, analyzing factors that modulate these metrics in both people with type 1 diabetes (T1D) or T2D.

## Materials and Methods

### Study design and aims

This is a retrospective analysis of real-world de-identified data from users of the FreeStyle Libre^®^ Continuous Glucose Monitoring System (Abbott Diabetes Care, Alameda, CA) collected from 2014 to 2021. The aim was to evaluate TITR and TIR glucose metrics in four groups: those self-identified to have T1D (multiple daily injection [MDI] and pump users), T2D on basal-bolus insulin therapy (MDI and pump users), T2D on basal insulin only, and T2D not on insulin treatment.

In 2019, users of the desktop reporting software—only compatible with the reader device, not the smartphone app—were prompted to complete an optional online questionnaire to collect self-reported information about the age category (reported in multiyear ranges), gender, type of diabetes, diabetes duration, and diabetes treatment. Those indicating T1D using basal-bolus insulin therapy (either MDI or continuous subcutaneous insulin infusion [CSII]), as well as persons with T2D using basal-bolus insulin therapy (either MDI or CSII), basal insulin or a noninsulin treatment, were included in the analysis. The reader's 90-day memory was de-identified and uploaded to a database when connected to personal computer-based reporting software with an internet connection. The software includes an agreement that de-identified data will be collected, allowing the users to agree to the collection and analysis of the data.

### Outcomes

The key CGM glycemic metrics^2,6^ were calculated for each individual 14-day sensor with at least 5 days of data collected. Summary of the trends of TIR and TITR by diabetes treatment group was determined in each of the 4 groups by 10 equal-sized groups—or decile groups—of average glucose (AG). For each decile group, the mean values of TITR and TIR were calculated, along with the corresponding mean values of glucose and glucose coefficient of variation (CV). In the T1D group, the subgroups of T1D duration were similarly summarized using 10 equal-sized bins by AG. Finally, in the T1D- and noninsulin-treated T2D groups, the TITR and TIR were determined across fixed incremental ranges of AG and glucose CV to evaluate in more detail the association of TITR and TIR with these metrics.

### Statistical analysis

The database was analyzed by structured query language routines, the Python programming language (www.python.org). The central tendencies of data were expressed as means and standard deviations.

Given the wide familiarity of A1C as a biomarker of average glycemia, the expected A1C was determined from AG^12–14^ to align the TIR and TITR findings with A1C-based thresholds related to prediabetes, T2 diabetes diagnosis, and T2 diabetes target A1C levels.^15,16^

## Results

### Patient characteristics

A total 667,567 sensors from 22,006 CGM users were available for analysis, with 13,213 users with T1 diabetes, 5937 users with T2 diabetes treated with basal-bolus insulin/pump, 1281 users with T2 diabetes treated with basal only insulin, and 1575 T2D users not on insulin therapies (Table 1). TIR and TITR of study groups and different subgroups are further detailed in Table 1.

**Table 1.** Characteristics of Continuous Glucose Monitoring Users Available for Analysis

	All	T1D	T2D, Basal-bolus and CSII	T2D, Basal	T2D, Non-insulin
*N*, Sensors	667,567	417,745	188,462	34,249	27,111
*N*, Users	22,006	13,213	5937	1281	1575
Male (%)	70	60	84	82	84
Most prevalent age category, years	55–64	55–64	65–74	65–74	55–64
Subjects with 1 or more micro- or macrovascular complication(s) (%)	34	24	54	48	32
Mean (SD) AG (mg/dL)	156 (32)	158 (32)	155 (31)	154 (33)	137 (31)
Mean (SD) CV (%)	34 (8)	37 (8)	29 (7)	28 (7)	25 (6)
Mean (SD) TIR (%)	66 (19)	62 (17)	71 (20)	71 (20)	83 (18)
Mean (SD) TITR (%)	43 (19)	41 (16)	44 (20)	45 (21)	60 (24)
CV groups (sensor count, percentage)					
16–20%	20,396 (3%)	2818 (1%)	10,570 (6%)	2280 (7%)	4728 (17%)
20–24%	60,151 (9%)	11,208 (3%)	34,803 (18%)	6302 (18%)	7838 (29%)
24–28%	95,330 (14%)	30,276 (7%)	49,739 (26%)	8755 (26%)	6560 (24%)
28–32%	114,394 (18%)	60,780 (15%)	42,397 (22%)	7396 (22%)	3821 (14%)
32–36%	119,286 (18%)	86,831 (21%)	26,021 (14%)	4727 (14%)	1707 (6%)
36–40%	104,611 (16%)	88,200 (21%)	13,303 (7%)	2477 (7%)	631 (2%)
40–44%	72,700 (11%)	65,151 (16%)	6104 (3%)	1177 (3%)	268 (1%)
44% and higher	77,690 (12%)	72,080 (17%)	4630 (2%)	766 (2%)	214 (1%)
Diabetes diagnosis duration (subject count, percentage)					
Less than a year	1159 (5%)	845 (6%)	97 (2%)	45 (4%)	172 (11%)
1–5 years	3658 (17%)	2577 (20%)	490 (8%)	127 (10%)	464 (30%)
6–10 years	3098 (14%)	1512 (12%)	971 (16%)	269 (21%)	346 (22%)
11–15 years	2994 (14%)	1254 (10%)	1205 (20%)	279 (22%)	256 (16%)
16–20 years	2600 (12%)	1136 (9%)	1116 (19%)	191 (15%)	157 (10%)
More than 20 years	8211 (38%)	5689 (44%)	2008 (34%)	353 (28%)	161 (10%)

AG, average glucose; CSII, continuous subcutaneous insulin infusion; CV, coefficient of variation; T1D, type 1 diabetes; T2D, type 2 diabetes; TTIR, time in tight range.

### The relationship between AG and TIR/TITR varies across different diabetes groups

**FIG. 1.**
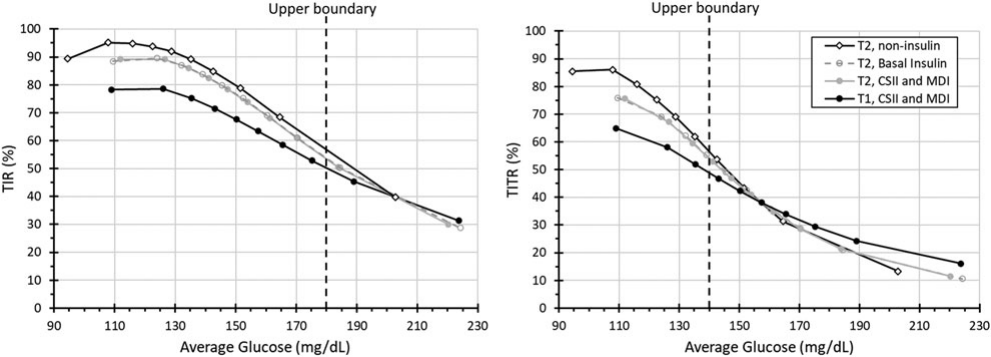
TIR (left) and TITR (right) in diabetes treatment groups according to equal-sized bins of AG. AG, average glucose; TIR, time in range; TITR, time in tight range.

The TIR and TITR in the 4 diabetes treatment groups are shown in Figure 1 in 10 equal-sized bins of AG (deciles). The span from the lowest to highest bin of AG is similar, but the noninsulin treated T2D group has lower values by 15–20 mg/dL. At a given AG between 110 and the upper bound of the range (180 or 140 mg/dL, respectively), the T2D noninsulin treatment group had the highest TIR and TITR values, associated with having the lowest glucose CV values (23–26%). The T2D insulin-treated groups (basal insulin, MDI, or insulin pump) had nearly identical TIR and TITR values with largely similar CVs (28–30%). The lowest TIR and TITR values at similar AG values was in the T1D group, associated with the highest CVs (36–38%).

Once AG reaches the upper bound of the range, TIR and TITR approach about 50% across all groups, regardless of glucose CV, and converge to ∼40% once AG is 20 mg/dL above the upper bound of the range. This is due to shape of the glucose distribution taking a bell-shaped curve and having about 50–60% of glucose values above range when AG is near the upper boundary. As the AG increases beyond the upper boundary of the target range, higher CV is associated with higher TIR and TITR (Supplementary Fig. S1). There is an alignment of TIR and TITR across the diabetes groups, but the association is affected by AG (especially when near the range boundaries) and glucose variability (Supplementary Fig. S2). The overall scatterplot of TIR and TITR is not well characterized by a line (*r*^2^ = 0.81, root-mean-square error = 8.1% TITR), with a complex curvilinear effect of CV (Supplementary Fig. S3A, B).

The T1D group was further evaluated by groups of diabetes duration, as shown in Supplementary Figure S4. Higher TITR and TIR values were found in those with fewer years of diabetes duration only when the AG was below the upper bound of the range (140 and 180 mg/dL, respectively). At these levels, those with less than five years duration had lower CV. At AG under 140 mg/dL, those with less than a year diabetes duration had lower glucose variability (CV = 29–33%) than those with one to five years duration (CV = 32–35%). For those with longer duration, all average CV values were 36% or higher. At higher AG levels above 140 mg/dL, even those recently diagnosed had CV values of 35% or more, and therefore, similar TITR and TIR values were observed across all the diabetes duration groups.

### Glycemic variability alters the relationship between AG and TIR/TITR

Given the important role of glycemic variability on the relationships between AG and TIR/TITR (described above) and the complex relationship between CV and TBR (Supplementary Fig. S5), data from the T1D and T2D noninsulin groups were analyzed in finer detail according to AG (95 mg/dL and higher in 5 mg/dL increments) and CV (16% and higher in 4% increments). Moreover, analysis of the correlation of TIR and TITR has shown this to be complex (Supplementary Fig. S3), and therefore, the relationship between these two glycemic markers requires additional analysis.

### AG and TIR/TITR relationships in T1D

**FIG. 2.**
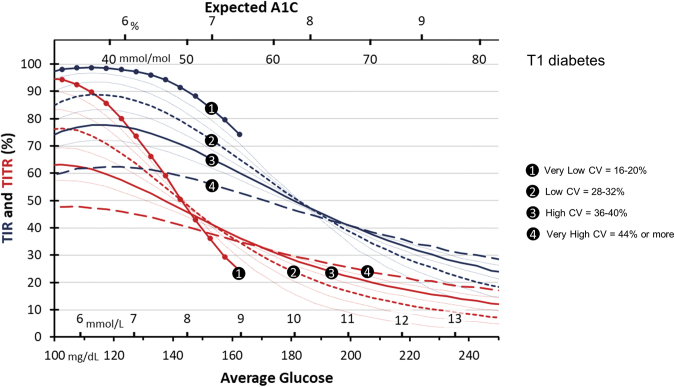
TIR (blue) and TITR (red) in individuals with T1D diabetes and different glucose CV across the range of AG and expected A1C. Solid line with circle markers: CV = 16–20%, Fine line: CV = 20–24%, Fine line: CV = 24–28%, Short Dashed line: CV = 28–32%, Fine line: CV = 32–36%, Solid line: CV = 36–40%, Fine line: CV = 40–44%, Long Dashed line: CV 44% or more. CV, coefficient of variation; T1D, type 1 diabetes.

The TIR and TITR across levels of AG—and therefore expected A1C—by CV groups for those with T1D is shown in Figure 2. The lowest CV group (16–20%) only occurred at AG = 165 mg/dL and below, while other CV groups spanned to higher AG levels. The lowest CV was the least frequent, with 1604 observations across the AG range and each 5 mg/dL bin of AG having at least 84 observations. The CV group ranging between 28% and 32% had 56,277 observations, with an average size of 1876 per AG bin, and a minimum size of 653 for AG values less than 200 mg/dL. The CV group ranging between 36% and 40% had 81,920 observations, with an average size of 3741 per AG bin, and a minimum size of 728 for AG values less than 200 mg/dL. The CV group of 44% or more had 65,290 observations, with an average size of 2976 per AG bin, and a minimum size of 956 for AG values less than 200 mg/dL.

At AG/A1C target of 154 mg/dL/7%, there are widely different TIR values at different levels of glucose CV; for the lowest CV group, TIR is 84% compared to only 56% in the highest CV group (28% difference). Since this AG is just above TITR range, the TITR values are found in a narrow range, between 38% and 42% (4% difference).

However, at lower AG/A1C levels of 112 mg/dL/5.6% both TIR and TITR have wide-ranging values according to glucose CV. For the lowest CV group, TIR is 99% compared to only 62% in the highest CV group (37% difference), while TITR shows even higher differences from 90% to 47%, respectively (43% difference). Conversely, as the AG increases beyond the upper boundaries of the TIR and TITR, higher glucose CV results in increased TIR and TITR.

### AG and TIR/TITR relationships in T2D

**FIG. 3.**
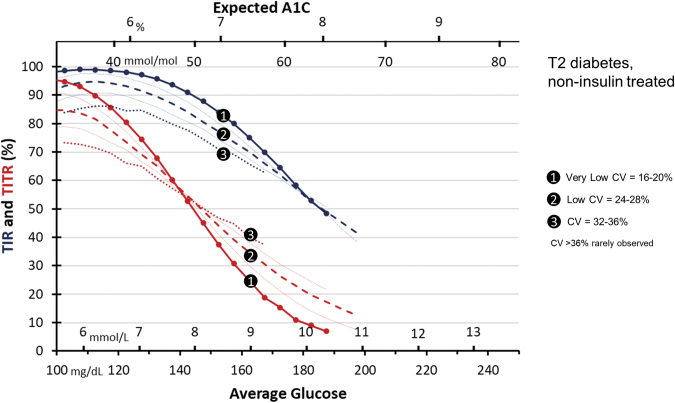
TIR (blue) and TITR ( red) in individuals with T2D on noninsulin therapies for different glucose CV across the range of AG and expected A1C. Solid line with circle markers: CV = 16–20%, Fine line: CV = 20–24%, Dashed line: CV = 24–28%, Fine line: CV = 28–32%, Dotted line: CV = 32–36%. T2D, type 2 diabetes.

TIR and TITR across levels of AG by CV groups for those with noninsulin-treated T2D is shown in Figure 3. The highest CV group (32–36%) was the least frequent, with 1330 observations across the AG range, each 5 mg/dL bin of AG had at least 60 observations. The CV group ranging between 24% and 28% had 6010 observations, with an average size of 301 per AG bin, and a minimum size of 51. The CV group of 16–20% had 3839 observations, with an average size of 240 per AG bin, and a minimum size of 52. In contrast to those with T1D, individuals with T2D not treated with insulin rarely had glucose CV above 36%, and therefore, were too sparsely observed to analyze. At the AG/A1C target of 154 mg/dL/7%, there are different TIR values as a function of glucose CV, though not as broad as the T1D group due to the narrower range of CV. For the very low CV = 16–20% group, TIR is 84% compared to 71% in the CV = 32–36% group. Similar to T1D, the TITR values are found in a narrow range, from 37% to 47%, given that the AG is just above the upper range of TITR. As shown above, as the AG increases beyond the upper boundary of the target range, those with higher glucose CV display higher TITR and TIR.

## Discussion

Our CGM data from a large cohort of individuals with T1D and T2D have yielded a number of observations including (1) higher glucose CV results in lower TIR and TITR for AG values <180 and <140 mg/dL, respectively, while increasing TIR and TITR for AG above these values, (2) glycemic variability has no effect on TIR and TITR at 180 and 140 mg/dL, respectively, (3) in general, the discordance between AG and TIR/TITR is less pronounced in T2D compared with T1D, attributed to lower glucose variability in the former group, and (4) TITR is more sensitive than TIR to changes in AG at near-normal glucose levels.

Across the groups analyzed in real-life settings, there was a wider range of glucose variability in T1D compared to T2D treated with insulin, followed by T2D not treated with insulin. These findings are generally consistent with recent analyses of TIR and TITR in clinical study participants with either T1D or T2D using basal-bolus insulin or AID^10^ and a study in youth with T1D,^11^ however, have a larger range of observed values across more individuals and those with T2D not using insulin. Our data are consistent with data from Passanisi et al. and the analysis by Beck et al. demonstrating that TIR–TITR relationship varies by CV, which we have further shown to display a complex relationship, given the dependence on AG. For a given TIR, the TITR can be higher for a higher CV, which is related to AG (Supplementary Figs. S2, and S3). For example, identical TIR of 68%, the noninsulin-treated T2D group (CV = 25%) has TITR at 31% and AG of 165 mg/dL, but in the T1D group (CV = 37%), TITR is 42% because AG is lower at 150 mg/dL. Beck et al. evaluated TBR in addition to CV, and while there is an association between TBR and CV, this relationship is complex and nonlinear (Supplementary Fig. S5), and therefore, a separate piece of work is required to investigate this area. However, given these data, it is likely that the observations of the interaction of TBR with the TIR-TITR would be consistent and those presented by Beck et al., and similar to the interaction of CV with the TIR–TITR relationship.

**FIG. 4.**
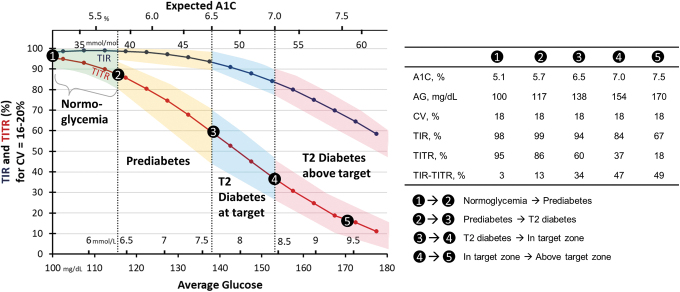
TIR (blue) and TITR (red) in the T2 noninsulin treated group at lowest glucose CV = 16–20% across the range of AG and expected A1C. Mean values and expected range are shown.

Data from healthy controls^7^ reported AG of 99 ± 7 mg/dL with CV at 17% ± 3%, thus close to the point labeled “1” in Figure 4. This region of AG/CV/TITR/TIR represents normoglycemia, where both TIR and TITR are above 95%. Increasing AG at constant CV defines a potential path of dysglycemia disease progression. As the expected A1C increases to the ADA definition of prediabetes^1^ of 5.7% and the diabetes range (A1C ≥6.5%), TITR is substantially reduced to below 86% and 60%, respectively. However, the TIR would still be above 98% and 93%, and therefore, difficult to distinguish from normoglycemia. In the lower glucose range—AG below 140 mg/dL—increased CV would result in lower values of TITR, and therefore, could be an effective clinical marker for early elevated glucose variability that would not be apparent from AG or A1C.

A likely contributor to the reduced variability and/or limited hypoglycemia in those not on insulin therapy is endogenous insulin production that controls excursions and limits large fluctuations in glucose levels. In individuals with T1D, glycemic variability was less pronounced in those with shorter diabetes duration, likely related to residual β-cell function in the early years following the diagnosis.^17^ Therefore, endogenous insulin production results in stronger associations between TIR/TITR and AG through lower glucose variability.

Achieving target TIR relies on normalizing glucose levels, while limiting hypoglycemic exposure. CGM T1D studies frequently demonstrate that average CV is over 40%,^18,19^ resulting in significant disconnect between A1C and TIR that can only be addressed by reducing glycemic variability. Importantly, TIR is sensitive to changes in CV when mean glucose is between 150 and 160 mg/dL, which is near the generally appropriate A1C target of 7%. However, at these glucose levels, higher CV is associated with increased TITR, giving the false impression of improved glucose control. In contrast, TITR becomes more sensitive to changes in CV when trying to bring glucose levels to near-normal ranges. In those with AG <140 mg/dL, TITR is more affected by AG and CV than TIR and hence reaching therapeutically acceptable TITR at lower AG values can only be achieved by keeping low CVs. Taken together, this intricate relationship between AG, CV, and TIR/TITR should be considered during the management of individuals with diabetes to appropriately optimize glucose control. From the practical point of view, our data provide new understanding of the ongoing role of TIR in managing patients T1D and T2D.

The findings from our work pose a crucial question: when should TITR be used in routine clinical practice? It appears TITR may be more important when normoglycemia is the clinical goal. By the rationale that the upper bound of the target range should be near but higher than the target AG, it stands that TITR will be more effective than TIR at observing changes in AG and glucose CV when AG is below 140 mg/dL. TITR is important because it better reflects near-normal, or healthy, glucose physiology than TIR. For example, our data indicate that it is perfectly possible to have A1c values <6.5% and TIR >90%, which would be regarded as good glycemic control, but with relatively low TITR at 60%. In such a scenario, TITR becomes a key marker for optimization of glycemic control to reach near-normal levels. Such tight control is the goal in some individuals with diabetes, including pregnant women and those diagnosed at a young age, given the long-life expectancy and the importance of metabolic memory.^20^ Moreover, the data in this study were in the range typically associated with the prediabetes range and even normoglycemia range. This indicates an important, separate role for TITR in patient management, including the capture of early stages of dysglycemia progression to prediabetes to diabetes. This may help in formulating new strategies for diabetes remission by targeting very early dysglycemia thus preventing not only diabetes but also prediabetes.

The strength of these data is having a large number of patients with different types of diabetes and therapies and across a wide variety of glucose profiles. The limitations of this analysis include the observational nature of the data collection, the fact that patient characteristics were self-reported, and the inability to probe into more specific subgroups due to the limited clinical data collected. Repeated measurements were not accounted for in analyses of linear regression, correlation, and smoothed line analyses, therefore, may overestimate the strength of some associations. Further collection of TIR and TITR in diverse groups are still needed, including those at early stages of dysglycemia.

## Conclusions

TIR and TITR are important glycemic markers that support shared decision-making with patients to address glycemic management goals. This real-world data analysis illustrates the broad scenarios expected to be encountered in clinical practice for the management of T1 and T2 diabetes, and how TIR and TITR have complementary roles with each covering specific clinical scenarios and individualized patient targets. TIR continues to be appropriate—and superior to TITR—to assess and guide management for those aiming to achieve a target of 7% A1C (and therefore, AG of 150–160 mg/dL). At these glucose levels and higher, TIR is sensitive to improvements in both AG and glucose CV, which are mutually beneficial for patients to manage overall glycemic exposure and potential hypoglycemia risk. In this clinical situation of target AG of 150–160 mg/dL, using TITR is problematic because it will increase with higher glucose CV—the incorrect means to “improve” TITR. Further, TITR will be below 50% until AG is below the upper range bound (140 mg/dL), which is substantially lower than the typical target of 150–160 mg/dL. In individuals requiring low AG and A1C targets, for instance, aiming for T2D remission or near-normoglycemia in T1D, it would be appropriate to use TITR to guide therapy. Importantly, TITR may be preferable to TIR when striving for A1C targets below 7% closer to normoglycemia, such as 6.5% or 6.0%, where TIR is insensitive to changes in AG and glucose CV. TITR helps patients be aware of and visualize additional excursions above target range compared to TIR. In the case of gestational diabetes and existing diabetes management during pregnancy, further data are needed to understand the optimal glycemic target ranges, currently recommended to be 63–140 mg/dL and recently referred to as time in pregnancy range.^21^ There may even be a need for a designation of time in tight pregnancy range, such as 63–120 mg/dL. Our understanding continues to evolve on how to set most appropriately CGM-based glycemic management targets, such as TIR and TITR, to assist with shared decision-making and to optimize and personalize glycemic management.

## Supplementary Material

Supplemental data

Supplemental data

Supplemental data

Supplemental data

Supplemental data
