# Supplementary material for: Is It Time to Move Beyond TIR to TITR? Real-World Data from Over 20,000 Users of Continuous Glucose Monitoring in Patients with Type 1 and Type 2 Diabetes
Source: Diabetes Technol Ther. 2024 Feb 1;26(3):203–10. doi: 10.1089/dia.2023.0565 (PMC10877396; doi:10.1089/dia.2023.0565)

Supplemental Figure 1. Comparison of glucose distributions with same average glucose, AG=190 mg/dL, but different coefficient of variation (CV), with the counter-intuitive effect of higher time in tight range (TITR) and time in range (TIR) in the case of higher glucose variability.


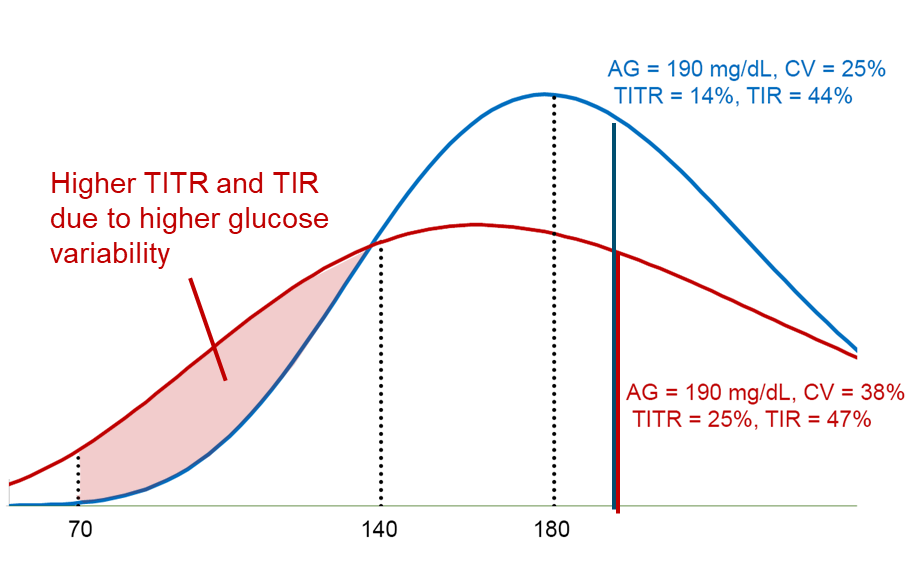

Supplement: Supplemental data [file Suppl_FigS1.docx]
