# Supplementary material for: Is It Time to Move Beyond TIR to TITR? Real-World Data from Over 20,000 Users of Continuous Glucose Monitoring in Patients with Type 1 and Type 2 Diabetes
Source: Diabetes Technol Ther. 2024 Feb 1;26(3):203–10. doi: 10.1089/dia.2023.0565 (PMC10877396; doi:10.1089/dia.2023.0565)

Supplemental Figure 3A. Scatterplot of time in tight range (TITR) and time in range (TIR) (n=667,567) with coefficient of variation (CV) of 25-25.5% in blue and CV=37-37.5% in red. Green line is linear regression. RMSE: Root mean square error.


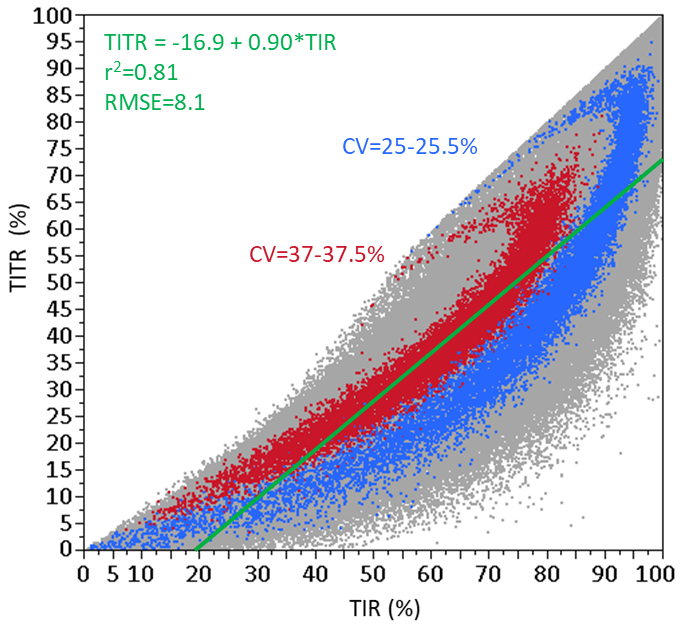


Supplemental Figure 3B. Scatterplot of time in range (TIR) and time in tight range (TITR) (n=667,567) with color-coded across the range of CV from 15-50%, respectively. Black line is smoothed mean value, purple line is linear regression. RMSE: Root mean square error.


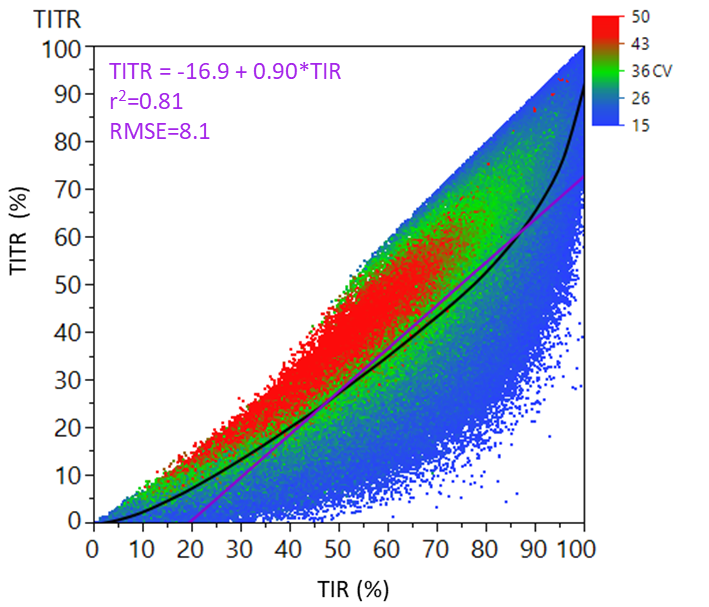

Supplement: Supplemental data [file Suppl_FigS3.docx]
