# Supplementary material for: Is It Time to Move Beyond TIR to TITR? Real-World Data from Over 20,000 Users of Continuous Glucose Monitoring in Patients with Type 1 and Type 2 Diabetes
Source: Diabetes Technol Ther. 2024 Feb 1;26(3):203–10. doi: 10.1089/dia.2023.0565 (PMC10877396; doi:10.1089/dia.2023.0565)

Supplemental Figure 4. Time in range (TIR, left) and time in tight range (TITR, right) in the T1 diabetes group by years since diabetes diagnosis according to equal-sized bins of average glucose.


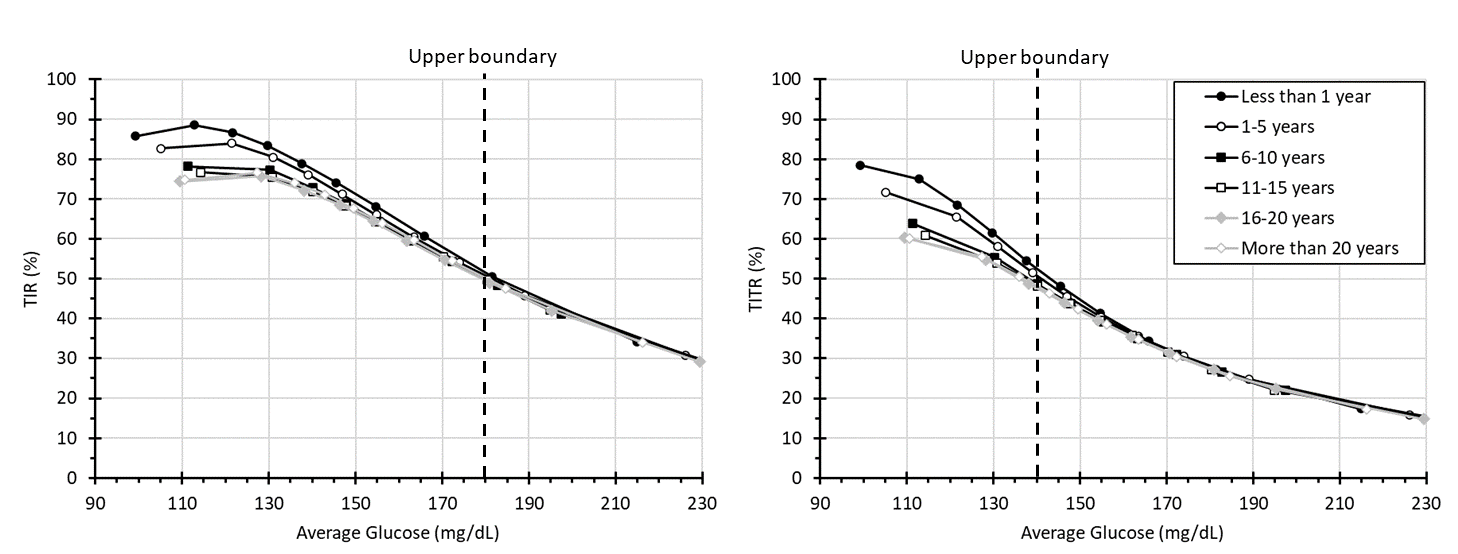

Supplement: Supplemental data [file Suppl_FigS4.docx]
