# Supplementary material for: Is It Time to Move Beyond TIR to TITR? Real-World Data from Over 20,000 Users of Continuous Glucose Monitoring in Patients with Type 1 and Type 2 Diabetes
Source: Diabetes Technol Ther. 2024 Feb 1;26(3):203–10. doi: 10.1089/dia.2023.0565 (PMC10877396; doi:10.1089/dia.2023.0565)

Supplemental Figure 5. Association between time below range (TBR, < 70 mg/dL) and coefficient of variation (CV) (n=667,567) shown as 10-level contour plot. Black line is smoothed mean value, green line is linear regression. RMSE: Root mean square error.


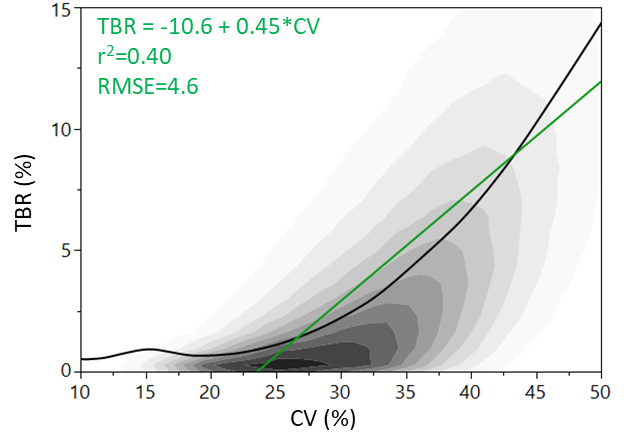

Supplement: Supplemental data [file Suppl_FigS5.docx]
